# Low skeletal muscle mass predicts melanoma-specific survival in melanoma patients treated with adjuvant immune checkpoint blockade

**DOI:** 10.1007/s00432-024-05812-4

**Published:** 2024-05-25

**Authors:** Miriam Mengoni, Andreas Dominik Braun, Mattes Simon Hinnerichs, Anar Aghayev, Thomas Tüting, Alexey Surov

**Affiliations:** 1 Department of Dermatology, University Hospital Magdeburg, Leipziger Straße 44, 39120 Magdeburg, Germany; 2 Department for Radiology and Nuclear Medicine, University Hospital Magdeburg, Magdeburg, Germany; 3 Department of Radiology, Neuroradiology and Nuclear Medicine, Johannes Wesling University Hospital, Ruhr University Bochum, Bochum, Germany

**Keywords:** Immunotherapy, Body composition, Low skeletal muscle mass, Melanoma, Adjuvant therapy

## Abstract

**Purpose:**

Adjuvant immunotherapy with immune checkpoint blockade(ICB) has greatly reduced the risk of recurrence and metastatic spread in early and advanced melanoma. However, not all patients benefit from adjuvant treatment: many patients show disease recurrence despite therapy, while those without recurrence harbor the risk for potentially irreversible adverse events. Biomarkers to select patients benefitting most from adjuvant therapy are currently lacking. As body composition assessment using CT images has shown promising results as a prognostic biomarker in stage IV melanoma, we aim to study the applicability of body composition parameters also in adjuvant melanoma treatment.

**Methods:**

We analyze body composition features via CT scans in a retrospective cohort of 109 patients with resected stage IIB-IV melanoma receiving an adjuvant first-line treatment with ICB in our department. In this analysis, we focus on the impact of body composition, especially the presence of low skeletal muscle mass (LSMM), on patients’ survival and occurrence of adverse events (AEs).

**Results:**

In uni- and multivariate analyses, we identify an association between CT-measured LSMM and melanoma-specific survival in patients treated with adjuvant ICB. Furthermore, LSMM is associated with a lower risk for therapy-related AEs, especially hypothyroidism, fatigue, and xerostomia. Conventional serological biomarkers e.g. S100 and LDH and measures of adipose tissue compartments did not show a correlation with survival or the occurrence of AEs.

**Conclusions:**

LSMM constitutes a novel biomarker for melanoma-specific survival in patients treated with adjuvant ICB.

**Supplementary Information:**

The online version contains supplementary material available at 10.1007/s00432-024-05812-4.

## Introduction

The success of immune checkpoint blockade (ICB) for patients with unresectable or metastatic melanoma has promoted the use of these agents also as adjuvant therapies for melanoma. Several clinical trials have successfully demonstrated the efficacy of PD1-based ICB, leading to the approval of nivolumab and pembrolizumab for the adjuvant treatment of melanoma, and the establishment of these therapies as the current standard of care (Eggermont et al. 2022; Larkin et al. 2023). The introduction of adjuvant ICB in increasingly early stages of disease has drastically increased the number of patients eligible for therapy (Luke et al. 2022; Kirkwood et al. 2023). Moreover, early-stage patients harbor a smaller individual risk for disease recurrence or progression, while the risk for irreversible adverse events (AEs) remains comparable to patients with advanced disease (Robert et al. 2019; Larkin et al. 2019). This inherently unfavorable risk-reward ratio of adjuvant therapy highlights the clinical need to identify patients who benefit most from ICB. However, despite this clinical need, biomarkers established in routine patient care are currently lacking.

Alterations of the physical constitution including syndromes such as cachexia have been described as common events in patients with solid cancers (Argilés et al. 2023). Furthermore, connections between body composition and response to modern treatments have been described in a pooled meta-analysis of melanoma patients (McQuade et al. 2018). In this work, adipose patients harboring a body mass index (BMI)  > 25 showed an improved survival under treatment with ICB. This effect was most pronounced in male patients with mild obesity (Naik et al. 2019). Interestingly, a high serum creatinine serving as a surrogate parameter for skeletal muscle mass, was also associated with longer survival (Naik et al. 2019). These results indicate a profound impact of body composition on treatment responses in patients with metastatic disease. How these results translate to the adjuvant treatment of melanoma is currently unknown.

The clinical interest in body composition has catalyzed the development of morphometric measures to quantify different tissue compartments as potential biomarkers of treatment response or occurrence of AEs. Due to the availability of CT scans in routine patient care, methods have been developed to facilitate accurate measurements of tissue compartments such as muscle mass and adipose tissue from single CT slices (Zopfs et al. 2020). In early studies, the prognostic value of sarcopenia as the reduction of muscle mass on CT images has been demonstrated in patients with respiratory or gastrointestinal solid tumors (Prado et al. 2008). Furthermore, visceral and subcutaneous adipose tissue abundance have been identified as favorable prognostic factors in melanoma patients with metastatic disease (Lee et al. 2022; Mengoni et al. 2023), further highlighting the use of radiologic measures of body composition as biomarkers. Again, the applicability of these results for patients in an adjuvant setting receiving ICB remains unknown.

In the current work, we therefore assess the association of body composition on outcome of a representative cohort of melanoma patients receiving ICB and analyze the impact of body composition on the occurrence of AEs.

## Materials and methods

### Study cohort selection

This retrospective study was approved by the institutional ethics committee of the Otto-von-Guericke University Magdeburg (Approval number 145/21, Ethics Committee, Otto-von-Guericke University Magdeburg, Germany). All patients with resected, stage IIB-IV melanoma treated with PD1-based adjuvant immunotherapy as first-line treatment at the Dermatology Department of the University Hospital Magdeburg from the years 2014 until 2023 were retrospectively assessed. All patients included into the present study underwent contrast-enhanced CT scans at baseline diagnosis in a time course of maximum 120 days before treatment initiation. Patients with prior melanoma-related therapies (except adjuvant interferon-alpha), secondary malignancies, no available CT staging at baseline as well as prior or concurrent immunosuppression were excluded from this trial. All patients received treatment until recurrence and/or disease progression according to the Response Evaluation Criteria in Solid Tumor (RECIST 1.1) criteria (Eisenhauer et al. 2009) or occurrence of inacceptable toxicity. All patients, including those who discontinued therapy, were included in the analysis to prevent attrition bias.

### Image acquisition

All CT images were acquired pretherapeutically on a multidetector CT scanner (Siemens Somatom Definition AS+, Siemens Healthineers, Germany, or Canon Aquilion Prime, Canon Medical Systems Corporation, Japan). Imaging was performed in supine position of patients with a standardized CT protocol (acquisition slice thickness of 1 mm with 5 mm reconstructions, tube voltage 120 kV with automatic tube current modulation, pitch factor 1.2, collimation 0.6 mm, application of 90 mL i.v. contrast medium (300 ml/mg Accupaque, GE Healthcare, USA)). All CT scans analyzed in this study were acquired in the portal venous phase.

### Segmentation and radiologic measurements of body composition parameters

All images were analyzed by trained radiologists who were blinded to the clinical course of the patients. For analysis, images were viewed in the soft tissue window. Total, subcutaneous, intermuscular and visceral adipose tissue as well as skeletal muscle cross-sectional areas were assessed semiautomatically on the height of the mid third lumbar vertebra (L3) using standard Hounsfield unit ranges (adipose tissue: − 190 to − 30 HU: skeletal muscle:− 29 to + 150 HU) (Richards et al. 2012). Tissue area and density were measured using ImageJ (version 1.48). Representative images describing the segmentation procedure are shown in Supplementary Fig.1. The skeletal muscle index (SMI) was calculated as the ratio of smooth muscle area (SMA) and the body height squared (muscle area/height^2^). In our current work, we assessed low skeletal muscle mass (LSMM) CT-based with the SMI threshold proposed by Prado et al.: < 52.4 cm^2^/m^2^ for male patients and < 38.5 cm^2^/m^2^ for female patients (Prado et al. 2008). The visceral to subcutaneous fat ratio (VSR) was obtained by dividing the visceral adipose tissue area with the subcutaneous adipose tissue area. For the VSR, a threshold of 1.1 was used. Adipose tissue gauge indices were calculated as described previously by our group (tissue area * tissue density/patient height) (Mengoni et al. 2023).

### Clinical data collection

Demographic and treatment data were compiled from electronic medical records. The following parameters were examined and included in the analysis: age, sex, body weight, height, tumor stage according to the American Joint Committee on Cancer Staging system (8th edition) (Gershenwald et al. 2017), laboratory parameters (lactate dehydrogenase (LDH), S100) and therapeutic agents used. As cutoff-values for S100 and LDH, the upper reference levels supplied by the assay manufacturer (0.11 µg/l for S100 and 3.75 µmol/s*l for LDH) were used. Additionally, dates of first dosing, disease recurrence and/or progression, death, cause of death and last follow-up were assembled to calculate the recurrence-free survival (RFS) and melanoma-specific survival (MSS) as primary outcome parameters of this study.

### Statistical analysis

Kaplan–Meier estimators were utilized for univariate analysis, and statistical significance was tested using a logrank-test. Furthermore, multivariate Cox regression models were fitted and adjusted for age. Analyses were conducted for both MSS and RFS. Missing values were omitted from analyses. Statistical significance of differences for adverse events were tested using Fisher’s exact test. Analyses were performed using python with standard library modules and the packages lifelines and scipy. p-values < 0.05 were considered statistically significant.

## Results

### Study cohort characteristics

We enrolled 109 patients with resected, stage IIB-IV melanoma in the current analysis who initiated adjuvant ICB for one year in our department between 2014 and 2023. The cohort consisted of 61 male and 48 female patients with a median age of 63 years. 63 patients received pembrolizumab, 41 patients nivolumab and 5 patients received other PD1-based therapy regiments. Patients were stratified by the presence or absence of low skeletal muscle mass (LSMM) as defined by Prado et al. (Prado et al. 2008). Patients with and without LSMM differed significantly in body weight, whereas all other characteristics were comparable between the two subgroups. The median follow-up of our cohort was 32.7 months, and 31.5 months for patients without disease recurrence. The full cohort characteristics are shown in Table 1.

**Table 1:** Study cohort characteristics

Characteristic	Total (n = 109)	No LSMM (n = 67)	LSMM (n = 42)	p
Median age* [years]	63 (50–75)	58 (50–72)	70 (55–77)	0.16
Sex				0.23
Sex: male	61 (56.0%)	34 (50.7%)	27 (64.3%)	
Sex: female	48 (44.0%)	33 (49.3%)	15 (35.7%)	
Therapeutic agent				0.68
Therapeutic agent: Pembrolizumab	63 (57.8%)	38 (56.7%)	25 (59.5%)	
Therapeutic agent: Nivolumab	41 (37.6%)	25 (37.3%)	16 (38.1%)	
Therapeutic agent: other PD1-based	5 (4.6%)	4 (6.0%)	1 (2.4%)	
Stage at therapy initiation				0.62
N/A	1 (0.9%)	1 (0.9%)	0 (0.0%)	
Stage IIB	1 (0.9%)	1 (1.5%)	0 (0.0%)	
Stage IIC	3 (2.8%)	2 (3.0%)	1 (2.4%)	
Stage IIIA	11 (10.2%)	7 (10.6%)	4 (9.5%)	
Stage IIIB	30 (27.8%)	15 (22.7%)	15 (35.7%)	
Stage IIIC	55 (50.9%)	36 (54.5%)	19 (45.2%)	
Stage IIID	5 (4.6%)	4 (6.1%)	1 (2.4%)	
Stage IV	3 (2.8%)	1 (1.5%)	2 (4.8%)	
Median weight* [kg]	80 (69–93)	85 (72–93)	79 (66–87)	0.03
Median height* [cm]	172 (166–180)	170 (165–178)	172 (168–180)	0.17
Median S100* [µg/l]	0.057 (0.042–0.076)	0.059 (0.042–0.078)	0.056 (0.04–0.075)	0.43
Median LDH* [µmol/s*l]	3.38 (2.96–3.773)	3.4 (3.105–3.773)	3.35 (2.95–3.72)	0.79

Data are presented as absolute number of patients with percentage in parentheses, with the exception for marked values (*), where interquartile ranges are given in parentheses

*LDH* lactate dehydrogenase, *LSMM* low skeletal muscle mass, *N/A* not available

### The presence of low skeletal muscle mass is associated with reduced melanoma-specific survival

**Fig. 1:**
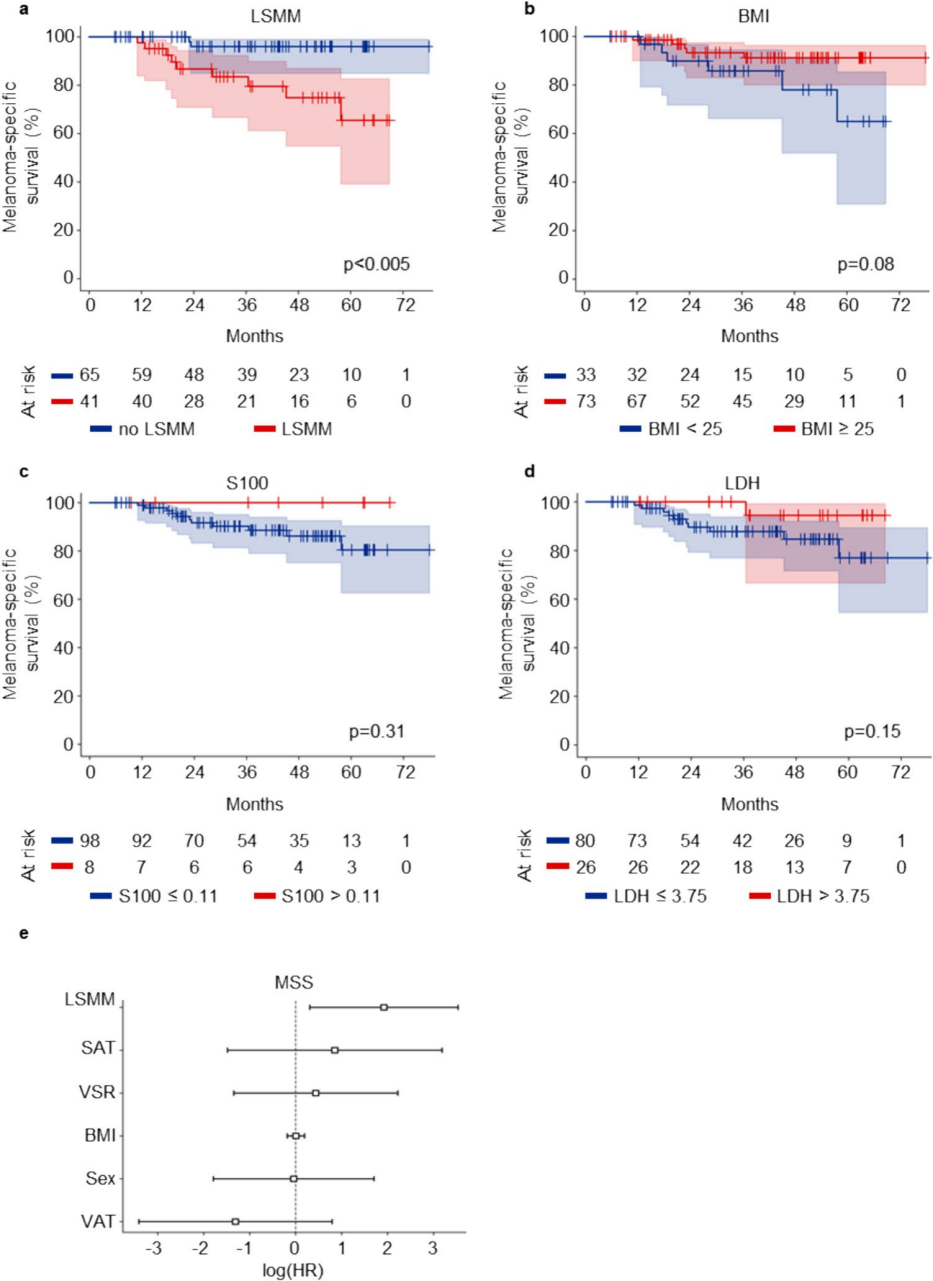
Low skeletal muscle mass outperforms conventional serological tumor markers as predictor of poor prognosis. **a**–**d** show Kaplan–Meier curves of melanoma-specific survival stratified by low skeletal muscle mass (**a**), BMI (**b**), S100 (**c**) and LDH (**d**). p-values were calculated by logrank test. **e** shows a multivariate regression analysis of body composition parameters and MSS. Each square represents the log(HR) for the coefficients, the whiskers denote the 95% confidence interval. *BMI* body mass index, *HR* hazard ratio, *LDH* lactate dehydrogenase, *LSMM* low skeletal muscle mass, *MSS* Melanoma-specific survival, *SAT* subcutaneous adipose tissue, *VAT* visceral adipose tissue, *VSR* visceral to subcutaneous fat ratio

We first analyzed the impact of LSMM on MSS. In our cohort, patients with LSMM revealed a significantly shorter MSS compared to patients without LSMM (Fig. 1a). Since obesity as measured by a BMI > 25 has been described to be associated with a favorable outcome of patients receiving ICB, we furthermore assessed the BMI in our cohort. While patients with a BMI > 25 showed a trend towards longer MSS, this effect was not statistically significant in a univariate analysis (Fig. 1b). Interestingly, the conventional serological biomarkers S100 and LDH failed to predict MSS in our cohort (Fig. 1c, d). A multivariate cox regression model for MSS substantiated the finding of LSMM as a predictor of worse outcome, revealing a hazard ratio of 6.82 (p = 0.02, Fig. 1e, Table 2). Other body composition measures were not associated with MSS (Supplementary Fig.2). In summary, these data identify LSMM as a novel biomarker for MSS, outperforming conventional biomarkers S100 and LDH in our adjuvant cohort.

**Table 2:** Multivariate cox regression model of the association between melanoma-specific or recurrence-free survival and body composition features

Characteristic	Melanoma-specific survival		Recurrence-free survival	
	Hazard ratio (95% CI)	p-value	Hazard ratio (95% CI)	p-value
LSMM	6.82 (1.36–34.28)	0.02	1.21 (0.59–2.51)	0.60
SAT	2.34 (0.23–24.20)	0.47	4.42 (0.55–35.53)	0.16
VSR	1.55 (0.26–9.23)	0.63	0.80 (0.35–1.82)	0.59
BMI	1.01 (0.83–1.21)	0.95	1.01 (0.92–1.11)	0.89
Sex	0.96 (0.17–5.50)	0.96	0.72 (0.32–1.61)	0.42
VAT	0.27 (0.03–2.21)	0.22	1.35 (0.46–3.95)	0.58

Shown are hazard ratios and p-values of a multivariate Cox regression model for MSS (left) or RFS (right). Both models were adjusted for age and sex

*BMI* body mass index, *CI* confidence interval, *LSMM* low skeletal muscle mass, *SAT* subcutaneous adipose tissue, *VAT* visceral adipose tissue, *VSR* visceral to subcutaneous fat ratio

### The presence of LSMM does not predict recurrence-free survival

**Fig. 2:**
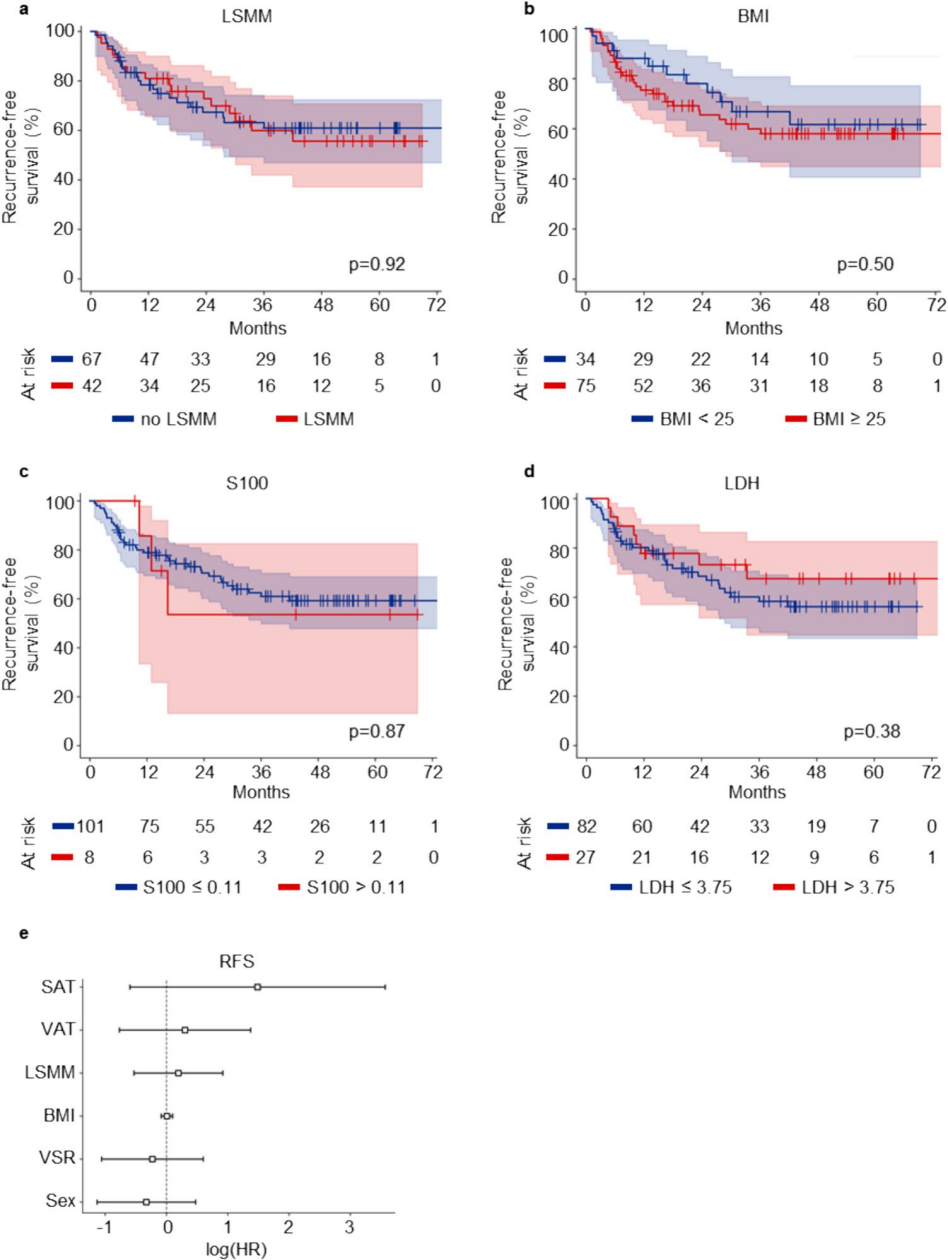
Low skeletal muscle mass does not predict recurrence-free survival (RFS). **a**–**d** show Kaplan–Meier curves of RFS stratified by low skeletal muscle mass (**a**), BMI (**b**), S100 (**c**) and LDH (**d**). p-values were calculated by logrank test. **e** shows a multivariate regression analysis of body composition parameters and RFS. Each square represents the log(HR) for the coefficients, the whiskers denote the 95% confidence interval. *BMI* body mass index, *HR* hazard ratio, *LDH* lactate dehydrogenase, *LSMM* low skeletal muscle mass, *RFS* recurrence-free survival, *SAT* subcutaneous adipose tissue, *VAT* visceral adipose tissue, *VSR* visceral to subcutaneous fat ratio

We furthermore assessed the relationship between the presence of LSMM and RFS. Whereas the RFS in our cohort was comparable to previously published results for melanoma patients treated with adjuvant ICB (Eggermont et al. 2022; Larkin et al. 2023), patients with LSMM surprisingly did not differ in their RFS compared to patients without LSMM in a univariate analysis of our cohort (Fig. 2a). Similarly, neither BMI nor the serological parameters S100 or LDH significantly predicted RFS in our patient cohort (Fig. 2b–d). In a multivariate cox regression model, no parameter reached statistical significance (Fig. 2e, Table 2). Again, no other body composition parameter was associated with RFS in our cohort (Supplementary Fig. 3).

### The presence of LSMM is associated with a lower risk of adverse events

Next, we investigated the association of body composition features with AEs in our cohort. We observed a total of 159 AEs in our cohort. Of these 82 events were of grade 1, 63 events of grade 2, 12 events of grade 3 and 2 events of grade 4. Interestingly, patients with LSMM harbored fewer occurrences of hypothyroidism, fatigue, and xerostomia (Table 3). The incidence of any AE or severe AEs of grade 3 or 4 did not significantly differ between patients with or without LSMM (any grade p = 0.68, grade 3–4 p = 0.25).

**Table 3:** Adverse events

Adverse event (AE)	Total	No LSMM	LSMM	p-value
Hypothyroidism	24.0 (22.0%)	17.0 (31.5%)	7.0 (12.7%)	0.0221
Fatigue	21.0 (18.3%)	15.0 (27.8%)	6.0 (10.9%)	0.030379
Hyperthyroidism	14.0 (12.8%)	10.0 (18.5%)	4.0 (7.3%)	0.093089
Pruritus	10.0 (9.2%)	5.0 (9.3%)	5.0 (9.1%)	1.000000
Diarrhea	10.0 (9.2%)	5.0 (9.3%)	5.0 (9.1%)	1.000000
Arthritis	9.0 (8.3%)	7.0 (13.0%)	2.0 (3.6%)	0.093269
Rash maculopapular	9.0 (8.3%)	3.0 (5.6%)	6.0 (10.9%)	0.489
Skin hypopigmentation	7.0 (6.4%)	4.0 (7.4%)	3.0 (5.5%)	0.716
ALAT increased	6.0 (5.5%)	3.0 (5.6%)	3.0 (5.5%)	1.000
Sarcoid-like reaction	6.0 (5.5%)	3.0 (5.3%)	3.0 (5.5%)	1.000000
Dry mouth, xerostomia	5.0 (4.6%)	5.0 (9.3%)	0.0 (0.0%)	0.027070
ASAT increased	5.0 (4.6%)	3.0 (5.3%)	2.0 (3.6%)	0.678654
Eczema	5.0 (4.6%)	2.0 (3.7%)	3.0 (5.5%)	1.000000
Colitis	4.0 (3.7%)	1.0 (1.9%)	3.0 (5.5%)	0.618023
Hyperglycemia	3.0 (2.8%)	2.0 (3.7%)	1.0 (1.8%)	0.618023
Pancreatitis	3.0 (2.8%)	2.0 (3.7%)	1.0 (1.8%)	0.618023
Pneumonitis	3.0 (2.8%)	1.0 (1.9%)	2.0 (3.6%)	1.000000
Dry skin	3.0 (2.8%)	2.0 (3.7%)	1.0 (1.8%)	0.618023
Arthralgia	2.0 (1.8%)	1.0 (1.9%)	1.0 (1.8%)	1.000000
Mucositis oral	2.0 (1.8%)	1.0 (1.9%)	1.0 (1.8%)	1.000000
Hypophysitis	1.0 (0.9%)	0.0 (0.0%)	1.0 (1.8%)	1.000000
Bronchial infection	1.0 (0.9%)	0.0 (0.0%)	1.0 (1.8%)	1.000000
Bullous dermatitis	1.0 (0.9%)	0.0 (0.0%)	1.0 (1.8%)	1.000000
Erectile dysfunction	1.0 (0.9%)	1.0 (1.9%)	0.0 (0.0%)	0.495413
Neutrophil count decreased	1.0 (0.9%)	1.0 (1.9%)	0.0 (0.0%)	0.495413
Headache	1.0 (0.9%)	1.0 (1.9%)	0.0 (0.0%)	0.495413
Pancreatic enzymes decreased	1.0 (0.9%)	1.0 (1.9%)	0.0 (0.0%)	0.495413
Alopecia	1.0 (0.9%)	0.0 (0.0%)	1.0 (1.8%)	1.000000

Shown are absolute number of events of adverse events (AEs) observed in the study, stratified also by the presence or absence of low skeletal muscle mass (LSMM). p-values shown are between cohorts of patients with and without LSMM as tested by Fisher’s exact test

*ALAT* alanine aminotransferase, *ASAT* aspartate aminotransferase, *LSMM* low skeletal muscle mass

## Discussion

In our current study, we describe the presence of LSMM as a risk factor for melanoma patients treated with adjuvant PD1-based ICB. To our knowledge, this is the first study examining the effect of body composition measures on the efficacy and tolerability of adjuvant ICB in melanoma patients of stages IIB-IV. Interestingly, pre-treatment LSMM was associated with a reduced MSS, whereas other parameters of body composition and routine serologic biomarkers (S100, LDH) did not correlate with patient outcome. At the same time, patients with LSMM less frequently experienced adverse events such as hypothyroidism under ICB. Since hypothyroidism and other immune-related adverse events have been described as favorable prognostic factors (Dawidowska et al. 2022), this further substantiates a potential immunosuppressive impact of LSMM. Previous reports have linked the presence of LSMM with poor prognosis also in stage III melanoma without adjuvant therapy (Sabel et al. 2011; Youn et al. 2022), suggesting a broader applicability of LSMM as a risk factor in melanoma. LSMM has also been identified as a predictor of worse outcome and increased frequency of adverse events in other cancer entities including patients with colon cancer receiving adjuvant chemotherapy (Jung et al. 2015). The mechanism how LSMM or other body composition features influence immune responses are currently unclear. Recent meta-analyses have observed sex-specific differences of body composition a predictor of improved outcome in melanoma under ICB, in which obesity served as a favorable indicator only in male, but not female patients (Trinkner et al. 2023). The interdependence between sex and body composition suggests that skeletal muscle and adipose tissue might alter hormonal responses governing anti-tumoral immune responses.

While the presence of LSMM predicted poor MSS in our cohort, we did not observe a difference for RFS in patients with LSMM. Similar discrepancies between PFS and overall survival have been described in meta-analyses of solid cancer clinical trials (Belin et al. 2020; Pasalic et al. 2020). These differences were not attributable to methodological errors, but rather caused by the longer post-progression survival enabled by novel therapies and additional second-line therapy options (Amir et al. 2012; Hess et al. 2019).

A major advantage of LSMM as a biomarker for melanoma is the simplicity of calculation, which is possible from routinely acquired CT images readily available for most patients. Automated systems assessing various body composition measures including LSMM are under active development (Lee et al. 2018; Graffy et al. 2019), further facilitating the applicability of our results into patient care. Moreover, the precision of body composition measurements might be further enhanced by deep-learning based volumetric models (Koitka et al. 2021).

The limited cohort size of our study poses a key limitation of our work. Moreover, the retrospective study design impedes the analysis of AEs since these are frequently underreported in retrospective analyses. Nonetheless, our results on adjuvant patients are consistent with reports from metastatic disease, substantiating the validity of our results. Further prospective trials using larger cohorts are required to fully uncover the usability of LSMM and other body composition parameters as predictors in adjuvant melanoma patients receiving ICB.

## Conclusion

We present the first study evaluating body composition features as biomarkers for melanoma patients treated with adjuvant ICB and identify LSMM as a predictive indicator of reduced melanoma-specific survival. LSMM was associated with a lower risk for AEs.

## Supplementary Information

Below is the link to the electronic supplementary material.

**Supplementary Figure 1: Body composition assessment. Exemplary description of the segmentation procedure. Shown are CT images on the height of the mid third lumbar vertebra of a patient with low skeletal muscle mass (LSMM) (a) and without LSMM (b). Greyscale image is shown left, corresponding segmentation results are shown right. The subcutaneous adipose tissue area is marked in blue, visceral adipose tissue area in yellow, intramuscular adipose tissue area in green and skeletal muscle area in red.** (TIF 4201 KB)

**Supplementary Figure 2: Univariate analysis of body composition parameters for melanoma-specific survival (MSS). Shown are Kaplan-Meier curves of melanoma-specific survival stratified by SAT, VAT, VSR, SATGI, VATGI, TATGI and IMATGI. p-values were calculated by logrank test. IMATGI, intermuscular adipose tissue gauge index, SAT, subcutaneous adipose tissue, SATGI, subcutaneous adipose tissue gauge index, TATGI, total adipose tissue gauge index, VAT, visceral adipose tissue, VATGI, visceral adipose tissue gauge index, VSR, visceral to subcutaneous fat ratio.** (TIF 2444 KB)

**Supplementary Figure 3: Univariate analysis of body composition parameters for recurrence-free survival (RFS). Shown are Kaplan-Meier curves of recurrence-free survival stratified by SAT, VAT, VSR, SATGI, VATGI, TATGI and IMAGTI. p-values were calculated by logrank test. IMATGI, intermuscular adipose tissue gauge index, SAT, subcutaneous adipose tissue, SATGI, subcutaneous adipose tissue gauge index, TATGI, total adipose tissue gauge index, VAT, visceral adipose tissue, VATGI, visceral adipose tissue gauge index, VSR, visceral to subcutaneous fat ratio.** (TIF 2472 KB)

## Data Availability

The datasets generated during and/or analyzed during the current study are available from the corresponding author on reasonable request.

## Abbreviations

AE: Adverse event
BMI: Body mass index
CI: Confidence interval
CT: Computed tomography
DLT: Dose-limiting toxicity
HR: Hazard ratio
ICB: Immune checkpoint blockade
IMATGI: Intermuscular adipose tissue gauge index
IQR: Interquartile range
LDH: Lactate dehydrogenase
LSMM: Low skeletal muscle mass
MSS: Melanoma-specific survival
PFS: Progression-free survival
RECIST: Response evaluation criteria in solid tumor
RFS: Recurrence-free survival
SAT: Subcutaneous adipose tissue
SATGI: Subcutaneous adipose tissue gauge index
SMI: Skeletal muscle index
SMA: Smooth muscle area
TAT: Total adipose tissue
TATGI: Total adipose tissue gauge index
VAT: Visceral adipose tissue
VATGI: Visceral adipose tissue gauge index
VSR: Visceral to subcutaneous fat ratio

## References

[CR1] Amir E, Seruga B, Kwong R et al (2012) Poor correlation between progression-free and overall survival in modern clinical trials: are composite endpoints the answer? Eur J Cancer 48:385–388. 10.1016/j.ejca.2011.10.028 22115991 10.1016/j.ejca.2011.10.028

[CR2] Argilés JM, López-Soriano FJ, Stemmler B, Busquets S (2023) Cancer-associated cachexia—understanding the tumour macroenvironment and microenvironment to improve management. Nat Rev Clin Oncol 20:250–264. 10.1038/s41571-023-00734-5 36806788 10.1038/s41571-023-00734-5

[CR3] Belin L, Tan A, De Rycke Y, Dechartres A (2020) Progression-free survival as a surrogate for overall survival in oncology trials: a methodological systematic review. Br J Cancer 122:1707–1714. 10.1038/s41416-020-0805-y 32214230 10.1038/s41416-020-0805-y PMC7250908

[CR4] Dawidowska A, Jagodzinska-Mucha P, Koseła-Paterczyk H et al (2022) Immune-related thyroid adverse events predict response to PD-1 blockade in patients with melanoma. Cancers 14:1248. 10.3390/cancers14051248 35267557 10.3390/cancers14051248 PMC8909092

[CR5] Eggermont AMM, Kicinski M, Blank CU et al (2022) Five-year analysis of adjuvant pembrolizumab or placebo in stage III melanoma. NEJM Evid 1:EVIDoa200214. 10.1056/EVIDoa2200214 10.1056/EVIDoa2200214 38319852

[CR6] Eisenhauer EA, Therasse P, Bogaerts J et al (2009) New response evaluation criteria in solid tumours: revised RECIST guideline (version 1.1). Eur J Cancer 45:228–247. 10.1016/j.ejca.2008.10.026 19097774 10.1016/j.ejca.2008.10.026

[CR7] Graffy PM, Liu J, Pickhardt PJ et al (2019) Deep learning-based muscle segmentation and quantification at abdominal CT: application to a longitudinal adult screening cohort for sarcopenia assessment. Br J Radiol 92:20190327. 10.1259/bjr.20190327 31199670 10.1259/bjr.20190327 PMC6724622

[CR8] Hess LM, Brnabic A, Mason O et al (2019) Relationship between progression-free survival and overall survival in randomized clinical trials of targeted and biologic agents in oncology. J Cancer 10:3717–3727. 10.7150/jca.32205 31333789 10.7150/jca.32205 PMC6636299

[CR9] Jung H-W, Kim JW, Kim J-Y et al (2015) Effect of muscle mass on toxicity and survival in patients with colon cancer undergoing adjuvant chemotherapy. Support Care Cancer off J Multinatl Assoc Support Care Cancer 23:687–694. 10.1007/s00520-014-2418-6 10.1007/s00520-014-2418-6 25163434

[CR10] Kirkwood JM, Del Vecchio M, Weber J et al (2023) Adjuvant nivolumab in resected stage IIB/C melanoma: primary results from the randomized, phase 3 CheckMate 76K trial. Nat Med 29:2835–2843. 10.1038/s41591-023-02583-2 37845511 10.1038/s41591-023-02583-2 PMC10667090

[CR11] Koitka S, Kroll L, Malamutmann E et al (2021) Fully automated body composition analysis in routine CT imaging using 3D semantic segmentation convolutional neural networks. Eur Radiol 31:1795–1804. 10.1007/s00330-020-07147-3 32945971 10.1007/s00330-020-07147-3 PMC7979624

[CR12] Larkin J, Chiarion-Sileni V, Gonzalez R et al (2019) Five-year survival with combined nivolumab and ipilimumab in advanced melanoma. N Engl J Med 381:1535–1546. 10.1056/NEJMoa1910836 31562797 10.1056/NEJMoa1910836

[CR13] Larkin J, Del Vecchio M, Mandalá M et al (2023) Adjuvant nivolumab versus ipilimumab in resected stage III/IV melanoma: 5-year efficacy and biomarker results from CheckMate 238. Clin Cancer Res off J Am Assoc Cancer Res 29:3352–3361. 10.1158/1078-0432.CCR-22-3145 10.1158/1078-0432.CCR-22-3145 PMC10472092 37058595

[CR14] Lee SJ, Liu J, Yao J et al (2018) Fully automated segmentation and quantification of visceral and subcutaneous fat at abdominal CT: application to a longitudinal adult screening cohort. Br J Radiol 91:20170968. 10.1259/bjr.20170968 29557216 10.1259/bjr.20170968 PMC6223139

[CR15] Lee JH, Hyung S, Lee J, Choi S-H (2022) Visceral adiposity and systemic inflammation in the obesity paradox in patients with unresectable or metastatic melanoma undergoing immune checkpoint inhibitor therapy: a retrospective cohort study. J Immunother Cancer 10:e005226. 10.1136/jitc-2022-005226 36002189 10.1136/jitc-2022-005226 PMC9413167

[CR16] Luke JJ, Rutkowski P, Queirolo P et al (2022) Pembrolizumab versus placebo as adjuvant therapy in completely resected stage IIB or IIC melanoma (KEYNOTE-716): a randomised, double-blind, phase 3 trial. Lancet 399:1718–1729. 10.1016/S0140-6736(22)00562-1 35367007 10.1016/S0140-6736(22)00562-1

[CR17] McQuade JL, Daniel CR, Hess KR et al (2018) Association of body-mass index and outcomes in patients with metastatic melanoma treated with targeted therapy, immunotherapy, or chemotherapy: a retrospective, multicohort analysis. Lancet Oncol 19:310–322. 10.1016/S1470-2045(18)30078-0 29449192 10.1016/S1470-2045(18)30078-0 PMC5840029

[CR18] Mengoni M, Braun AD, Hinnerichs MS et al (2023) Subcutaneous fat abundance and density are associated with an enhanced response to immunotherapy in metastatic melanoma: a retrospective cohort study. Acad Radiol. 10.1016/j.acra.2023.05.007 37331867 10.1016/j.acra.2023.05.007

[CR19] Naik GS, Waikar SS, Johnson AEW et al (2019) Complex inter-relationship of body mass index, gender and serum creatinine on survival: exploring the obesity paradox in melanoma patients treated with checkpoint inhibition. J Immunother Cancer 7:89. 10.1186/s40425-019-0512-5 30922394 10.1186/s40425-019-0512-5 PMC6440018

[CR20] Pasalic D, McGinnis GJ, Fuller CD et al (2020) Progression-free survival is a suboptimal predictor for overall survival among metastatic solid tumour clinical trials. Eur J Cancer 136:176–185. 10.1016/j.ejca.2020.06.015 32702645 10.1016/j.ejca.2020.06.015 PMC7702022

[CR21] Prado CMM, Lieffers JR, McCargar LJ et al (2008) Prevalence and clinical implications of sarcopenic obesity in patients with solid tumours of the respiratory and gastrointestinal tracts: a population-based study. Lancet Oncol 9:629–635. 10.1016/S1470-2045(08)70153-0 18539529 10.1016/S1470-2045(08)70153-0

[CR22] Richards CH, Roxburgh CSD, MacMillan MT et al (2012) The relationships between body composition and the systemic inflammatory response in patients with primary operable colorectal cancer. PLoS ONE 7:e41883. 10.1371/journal.pone.0041883 22870258 10.1371/journal.pone.0041883 PMC3411715

[CR23] Robert C, Ribas A, Schachter J et al (2019) Pembrolizumab versus ipilimumab in advanced melanoma (KEYNOTE-006): post-hoc 5-year results from an open-label, multicentre, randomised, controlled, phase 3 study. Lancet Oncol 20:1239–1251. 10.1016/S1470-2045(19)30388-2 31345627 10.1016/S1470-2045(19)30388-2

[CR24] Sabel MS, Lee J, Cai S et al (2011) Sarcopenia as a prognostic factor among patients with stage III melanoma. Ann Surg Oncol 18:3579–3585. 10.1245/s10434-011-1976-9 21822551 10.1245/s10434-011-1976-9

[CR25] Trinkner P, Günther S, Monsef I et al (2023) Survival and immunotoxicities in association with sex-specific body composition patterns of cancer patients undergoing immune-checkpoint inhibitor therapy—a systematic review and meta-analysis. Eur J Cancer Oxf Engl 1990 184:151–171. 10.1016/j.ejca.2023.01.030 10.1016/j.ejca.2023.01.030 36931074

[CR26] Youn S, Eurich DT, McCall M et al (2022) Skeletal muscle is prognostic in resected stage III malignant melanoma. Clin Nutr Edinb Scotl 41:1066–1072. 10.1016/j.clnu.2022.03.001 10.1016/j.clnu.2022.03.001 35397311

[CR27] Zopfs D, Theurich S, Große Hokamp N et al (2020) Single-slice CT measurements allow for accurate assessment of sarcopenia and body composition. Eur Radiol 30:1701–1708. 10.1007/s00330-019-06526-9 31776743 10.1007/s00330-019-06526-9

[CR28] Gershenwald JE, Scolyer RA, Hess KR et al (2017) Melanoma staging Evidence-based changes in the American Joint Committee on Cancer eighth edition cancer staging manual. CA Cancer J Clin 67:472–492. 10.3322/caac.21409 10.3322/caac.21409 PMC5978683 29028110

