# Supplementary figures and images for: Low skeletal muscle mass predicts melanoma-specific survival in melanoma patients treated with adjuvant immune checkpoint blockade
Source: J Cancer Res Clin Oncol. 2024 May 25;150(5):275. doi: 10.1007/s00432-024-05812-4 (PMC11127816; doi:10.1007/s00432-024-05812-4)

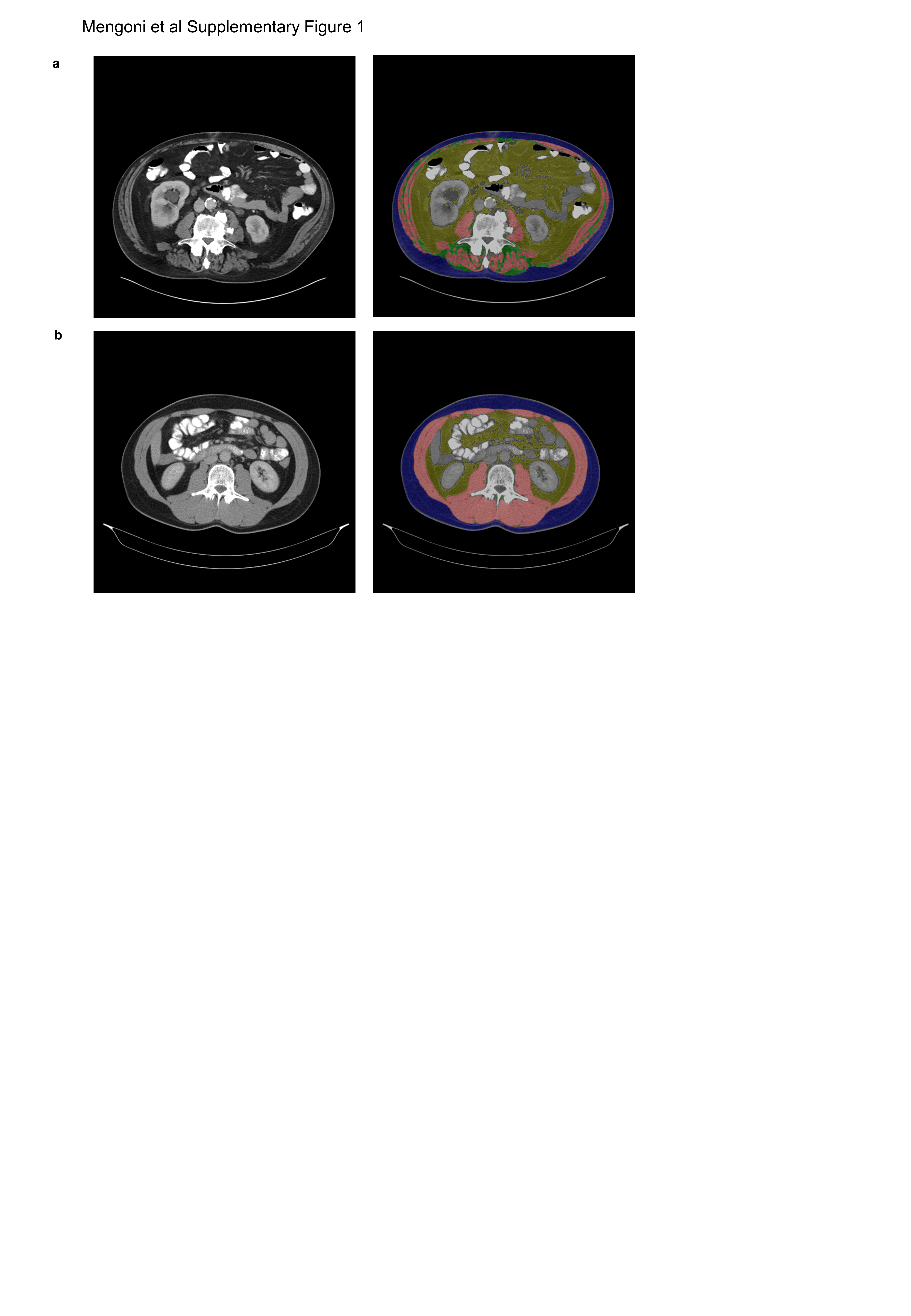

Supplement: Supplementary file 1 — Supplementary Figure 1: Body composition assessment. Exemplary description of the segmentation procedure. Shown are CT images on the height of the mid third lumbar vertebra of a patient with low skeletal muscle mass (LSMM) (a) and without LSMM (b). Greyscale image is shown left, corresponding segmentation results are shown right. The subcutaneous adipose tissue area is marked in blue, visceral adipose tissue area in yellow, intramuscular adipose tissue area in green and skeletal muscle area in red. (TIF 4201 KB) [file 432_2024_5812_MOESM1_ESM.tif]

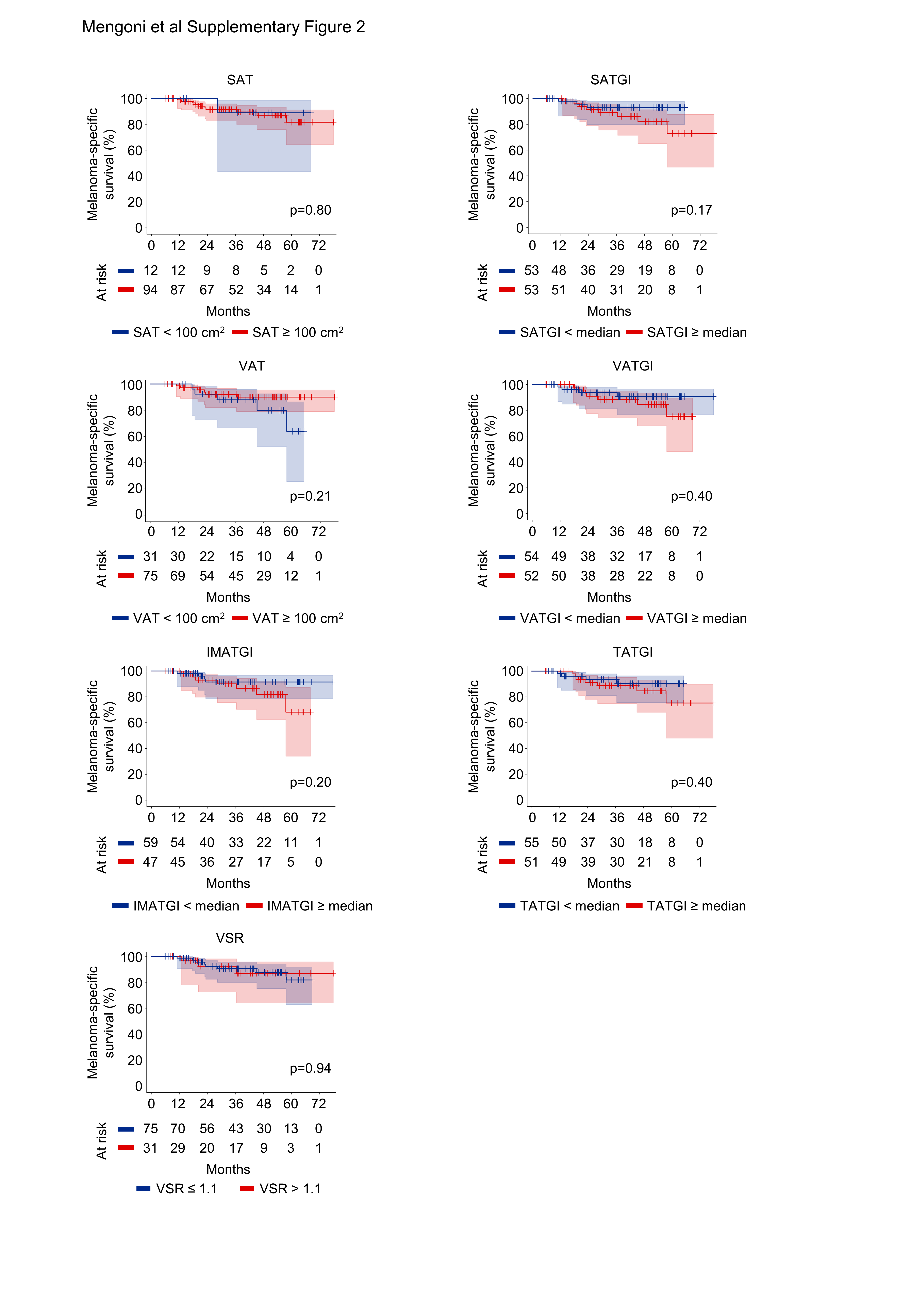

Supplement: Supplementary file 2 — Supplementary Figure 2: Univariate analysis of body composition parameters for melanoma-specific survival (MSS). Shown are Kaplan-Meier curves of melanoma-specific survival stratified by SAT, VAT, VSR, SATGI, VATGI, TATGI and IMATGI. p-values were calculated by logrank test. IMATGI, intermuscular adipose tissue gauge index, SAT, subcutaneous adipose tissue, SATGI, subcutaneous adipose tissue gauge index, TATGI, total adipose tissue gauge index, VAT, visceral adipose tissue, VATGI, visceral adipose tissue gauge index, VSR, visceral to subcutaneous fat ratio. (TIF 2444 KB) [file 432_2024_5812_MOESM2_ESM.tif]

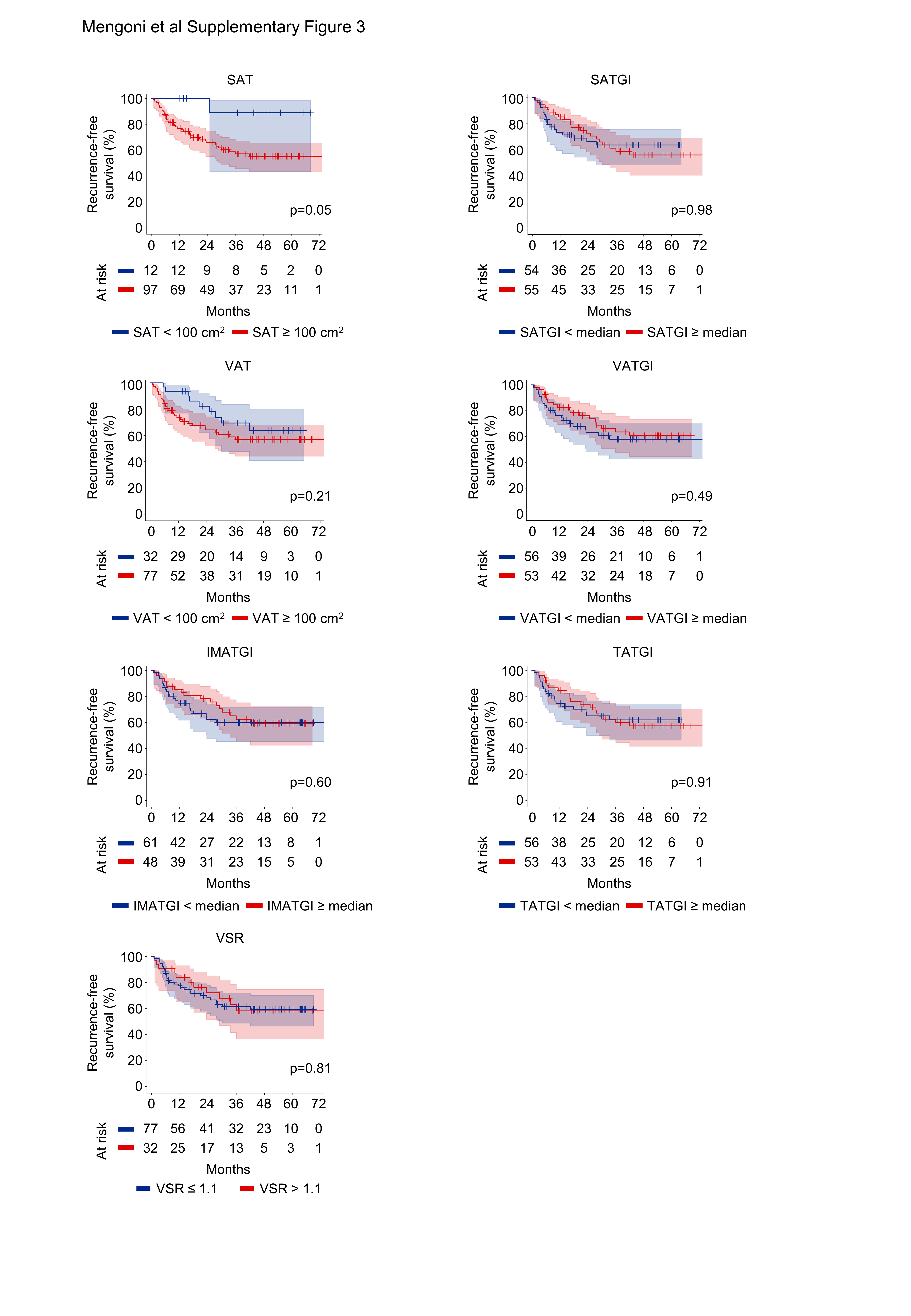

Supplement: Supplementary file 3 — Supplementary Figure 3: Univariate analysis of body composition parameters for recurrence-free survival (RFS). Shown are Kaplan-Meier curves of recurrence-free survival stratified by SAT, VAT, VSR, SATGI, VATGI, TATGI and IMAGTI. p-values were calculated by logrank test. IMATGI, intermuscular adipose tissue gauge index, SAT, subcutaneous adipose tissue, SATGI, subcutaneous adipose tissue gauge index, TATGI, total adipose tissue gauge index, VAT, visceral adipose tissue, VATGI, visceral adipose tissue gauge index, VSR, visceral to subcutaneous fat ratio. (TIF 2472 KB) [file 432_2024_5812_MOESM3_ESM.tif]
